# A retrospective analysis of paraquat and diquat poisoning: a single-center experience

**DOI:** 10.3389/fmed.2026.1796823

**Published:** 2026-05-08

**Authors:** Yuquan Chen, Yuqiang Lin, Yifan Ye, Meiwen Xie, Zhiqian Yang, Zhi Wang

**Affiliations:** Department of Occupational Diseases and Poisoning, Guangzhou Occupational Disease Prevention and Treatment Hospital (Guangzhou Twelfth People’s Hospital), Guangzhou, China

**Keywords:** diquat, paraquat, poisoning, pulmonary fibrosis, toxic encephalopathy

## Abstract

**Introduction:**

Paraquat (PQ) and diquat (DQ) are highly toxic bipyridyl herbicides, but their dominant organ injury patterns and clinical outcomes are not identical. This study aimed to compare the clinical characteristics, organ injury profiles, and prognosis of acute PQ and DQ poisoning.

**Methods:**

This retrospective single-center cohort study included consecutive patients with acute oral PQ or DQ poisoning treated at Guangzhou Occupational Disease Prevention and Treatment Hospital between January 2012 and January 2022. Baseline characteristics, clinical manifestations, admission laboratory findings, and in-hospital outcomes were compared between 270 patients with PQ poisoning and 115 patients with DQ poisoning.

**Results:**

PQ poisoning was associated with a higher crude in-hospital mortality rate than DQ poisoning (142/270 [52.6%] vs. 29/115 [25.2%], *p* < 0.001), shorter hospital stay, higher admission creatinine levels, and more frequent mediastinal emphysema. In contrast, DQ patients were younger, reported larger ingestion amounts, presented later to the local hospital, and more often showed nausea/vomiting, disturbance of consciousness, seizures, reduced responsiveness, agitation, dysarthria, anuria, and pleural effusion. The DQ group also had significantly higher CK and CK-MB levels, suggesting more prominent neuromuscular involvement. Kaplan–Meier analysis showed a significant difference in overall survival between the two groups.

**Conclusion:**

PQ and DQ poisoning show distinct clinical phenotypes and should not be managed as interchangeable toxic syndromes. Rapid toxin identification and toxin-specific monitoring may help guide early risk stratification and supportive care.

## 1 Introduction

Paraquat (1, 1′-dimethyl-4,4′-bipyridinium cation; PQ) and diquat (1, 1′-ethylene- 2,2′-bipyridinium ion; DQ) are nonselective, quick-acting bipyridyl herbicides that have been widely used in agricultural production (1–3). Both compounds are highly toxic to humans, and accidental or intentional ingestion may cause multiple organ injury and death even at relatively low doses (2–4). Although PQ and DQ share a similar chemical structure, their major target organs and clinical manifestations are not identical. PQ poisoning most prominently affects the lungs and is strongly associated with pulmonary fibrosis and respiratory failure (5–7). By contrast, DQ is not selectively concentrated in the lungs and is more often associated with central nervous system injury, rhabdomyolysis, and toxic encephalopathy in severe cases (2, 8–12). Although paraquat was banned in China on July 1, 2016, exposure to both PQ and DQ continues to be encountered in clinical practice. With the increasing use of diquat, reported DQ poisonings have also increased in recent years (1, 8, 9). Previous studies have largely focused on PQ or DQ poisoning separately, and direct clinical comparisons between the two toxins remain limited. Several retrospective cohort studies and prognostic model studies have highlighted the importance of organ injury patterns and early risk stratification in acute bipyridyl herbicide poisoning (5–9, 13–15). Therefore, this study aimed to compare the clinical characteristics, organ injury patterns, and prognostic outcomes of patients with acute PQ and DQ poisoning.

## 2 Materials and methods

### 2.1 Study design

This was a retrospective single-center cohort study of consecutive patients with acute PQ or DQ poisoning treated at Guangzhou Occupational Disease Prevention and Treatment Hospital (Guangzhou Twelfth People’s Hospital), Guangzhou, China, between 2012 and 2022. We compared baseline characteristics, clinical manifestations, admission laboratory findings, in-hospital outcomes, and organ injury patterns between patients with clinically confirmed PQ and DQ poisoning. In addition, for the revised analysis, we further reviewed the available records to better characterize the epidemiologic features of the cohort and the dominant fatal clinical pathways among non-survivors where documentation permitted.

### 2.2 Ethics statement

This retrospective study was approved by the Medical Ethics Committee of Guangzhou Occupational Disease Prevention and Treatment Hospital (Guangzhou Twelfth People’s Hospital). All medical records were reviewed in a de-identified manner, and access to study data was restricted to the investigators. Because this study used anonymized retrospective clinical data, the requirement for written informed consent was waived by the Medical Ethics Committee.

### 2.3 Criteria for case inclusion and exclusion

#### 2.3.1 Inclusion criteria

1 Consecutive patients with acute single oral ingestion of PQ or DQ who presented to a healthcare facility within 48 h after exposure.
2 Diagnosis supported by exposure history and toxicological testing of blood and/or urine.

#### 2.3.2 Exclusion criteria

1 Mixed poisoning with other pesticides or toxic agents.
2 Pre-existing severe chronic diseases that could substantially confound prognosis assessment (for example, advanced chronic kidney disease, decompensated chronic liver disease, or severe pre-existing neurologic disease).
3 Transfer to another facility before outcome ascertainment or incomplete key clinical records.

### 2.4 Data collection

#### 2.4.1 Basic information

We retrospectively analyzed all eligible patients admitted to Guangzhou Occupational Disease Prevention and Treatment Hospital (Guangzhou Twelfth People’s Hospital) after oral PQ or DQ ingestion between 2012 and 2022. Baseline variables included sex, age, estimated ingestion amount, time from exposure to presentation at the local hospital, treatment outcome, and treatment duration. Treatment time was defined as the length of hospital stay. Where available from the medical records, we also reviewed exposure-related information and the dominant fatal clinical pathway among non-survivors for the revised descriptive analysis. Patients transferred to other facilities before outcome evaluation and those with incomplete key clinical records were excluded.

#### 2.4.2 Clinical symptoms

Clinical manifestations were recorded at presentation, including gastrointestinal symptoms (nausea/vomiting, abdominal pain, and sore throat), neurologic symptoms (headache, unconsciousness, convulsions, seizures, dysphoria, dysarthria, limb weakness, blurred vision, unresponsiveness, cerebral hemorrhage, cerebral edema, and brain herniation), and respiratory symptoms (cough, shortness of breath, and lung exudation).

#### 2.4.3 Laboratory tests

Laboratory indices obtained at admission included white blood cell (WBC) count, alanine aminotransferase (ALT), aspartate aminotransferase (AST), blood glucose, creatine kinase (CK), CK-MB, serum creatinine (Cr), serum amylase (AMY), and PaO2/FiO2. Initial screening was performed using the urine sodium dithionite test, followed by blood and/or urine toxicology testing to confirm the specific toxin exposure. Several analytical approaches, including chromatographic methods, have been reported for the detection and quantification of PQ and DQ in human biological matrices, supporting the practical importance of toxin-specific confirmation in clinical and forensic settings (22, 25). In this cohort, the ingested paraquat and diquat products were typically 20% formulations.

#### 2.4.4 Treatment protocol

Patients were managed according to the routine clinical practice of our center after admission. Toxin elimination was initiated as early as possible and included gastric lavage, activated charcoal adsorption, mannitol-induced catharsis, furosemide-assisted diuresis, and blood purification when clinically indicated. Hemoperfusion was generally performed 1–2 times daily, preferably as early as possible, and was discontinued when blood/urine paraquat or urinary diquat testing became negative. To reduce target-organ injury associated with paraquat or diquat poisoning, methylprednisolone sodium succinate (80–120 mg, intravenous infusion, twice daily) was commonly administered. In patients with cerebral edema, dehydration therapy was given, including glycerol fructose and sodium chloride injection (25 g, intravenous infusion, twice daily), with albumin used alternately when necessary. Citicoline sodium injection (0.5 g, intravenous infusion, once daily) was used for neurotrophic support when indicated. Additional supportive therapies, such as hepatoprotective treatment, gastric protection, and other symptomatic measures, were provided according to the patients’ clinical conditions.

### 2.5 Statistical analysis

**Figure 1:**
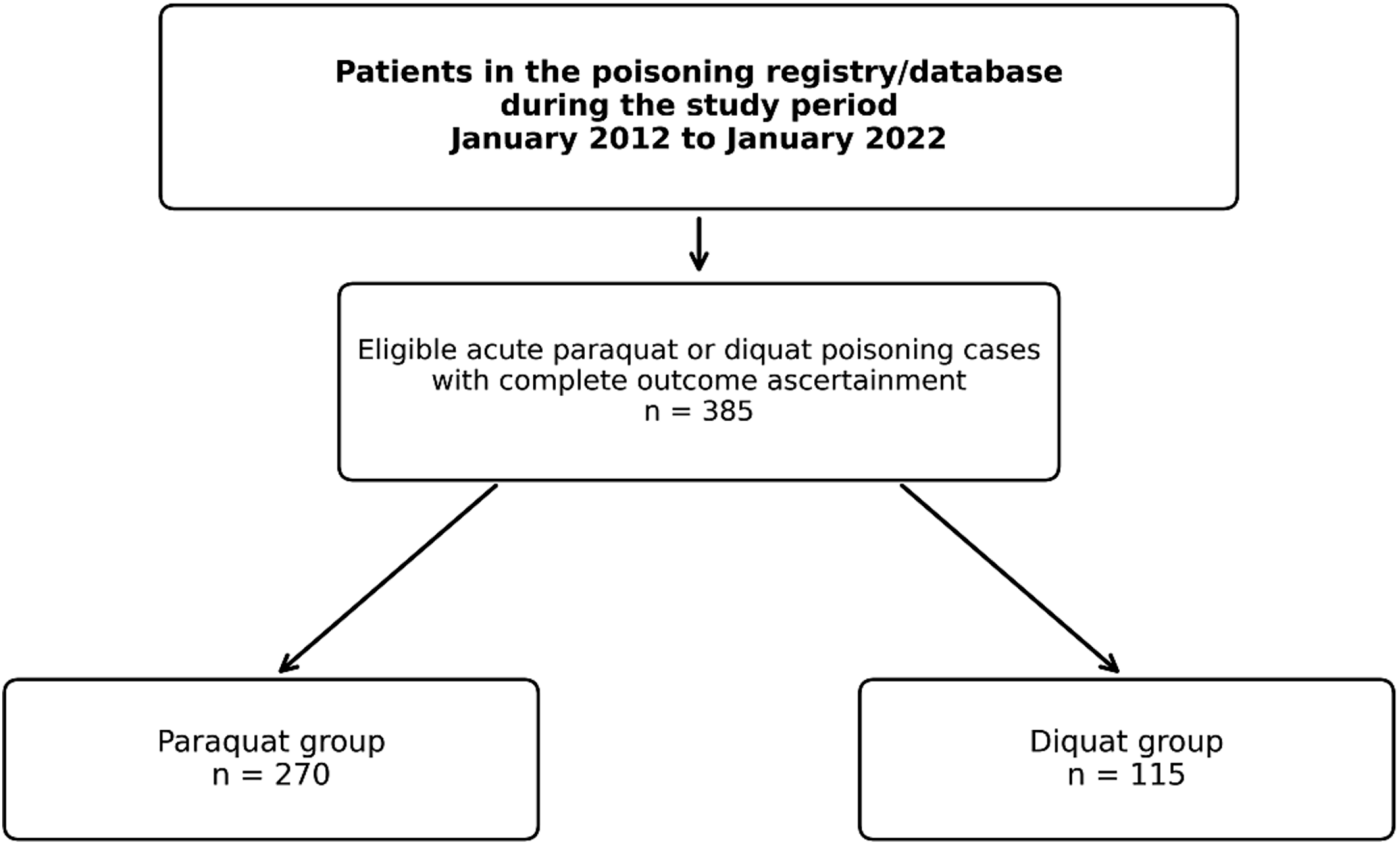
Flowchart of patient identification and final analytical cohort.

Continuous variables were assessed for normality using the Shapiro–Wilk test. Because most continuous variables in this cohort were non-normally distributed, they are presented as median (interquartile range [IQR]) and were compared using the Mann–Whitney U test. Categorical variables are presented as frequencies and percentages and were compared using the chi-square test or Fisher’s exact test, as appropriate. Survival was analyzed using the Kaplan–Meier method, and differences between groups were assessed with the log-rank test. All statistical analyses were performed using IBM SPSS Statistics version 24.0 and GraphPad Prism 10.1.2. A two-sided *p* value < 0.05 was considered statistically significant. Because this was a retrospective observational study including all consecutive eligible patients during the predefined study period, no formal *a priori* sample size calculation was performed. The study size was therefore determined by the total number of patients meeting the eligibility criteria during the study period, consistent with STROBE-oriented reporting for observational research (Figure 1).

## 3 Results

From 2012 to 2022, 385 patients were included in the study: 270 (70.1%) with acute PQ poisoning and 115 (29.9%) with acute DQ poisoning. Among all patients, 191 were male and 194 were female, and the median age was 26.0 years (IQR, 20.0–36.0; range, 11–80 years). All patients had a positive urine sodium dithionite screening result. Compared with the PQ group, patients in the DQ group were younger and had larger reported ingestion amounts, a longer time from exposure to presentation at the local hospital, and a longer hospital stay (all *p* ≤ 0.006). The crude in-hospital mortality rate was higher in the PQ group than in the DQ group (52.6% vs. 25.2%, *p* < 0.001). Admission WBC, ALT, AST, and amylase levels did not differ significantly between groups, whereas CK and CK-MB were higher in the DQ group and creatinine was higher in the PQ group (Table 1).

**Table 1:** Comparison of baseline characteristics, clinical manifestations, and admission laboratory findings between the PQ and DQ groups.

Variable	PQ (*n* = 270)	DQ (*n* = 115)	*P* value
Demographic and baseline characteristics, median (IQR) or *n* (%)			
Male sex, *n* (%)	135 (50.0)	56 (48.7)	0.902
Age, years	27.0 (21.0–38.0)	24.0 (18.5–29.0)	0.001
Ingestion amount, mL	20.0 (10.0–50.0)	40.0 (10.0–100.0)	0.003
Time from exposure to presentation at the local hospital, h	1.5 (0.6–3.0)	2.0 (1.0–6.0)	<0.001
Length of hospital stay, days	10.0 (2.0–16.0)	14.0 (3.0–21.0)	0.006
Clinical outcomes and manifestations, *n* (%)			
In-hospital death, *n* (%)	142 (52.6)	29 (25.2)	<0.001
Nausea/vomiting	102 (37.8)	82 (71.3)	<0.001
Oropharyngeal mucosal erosion	88 (32.6)	39 (33.9)	0.894
Sore throat	168 (62.2)	70 (60.9)	0.892
Abdominal pain	76 (28.1)	32 (27.8)	1.000
Dizziness/headache	37 (13.7)	30 (26.1)	0.005
Cough/sputum production	31 (11.5)	18 (15.7)	0.339
Chest tightness	47 (17.4)	22 (19.1)	0.796
Shortness of breath	34 (12.6)	9 (7.8)	0.237
Dyspnea	26 (9.6)	8 (7.0)	0.516
Limb weakness	28 (10.4)	21 (18.3)	0.050
Oliguria	10 (3.7)	10 (8.7)	0.077
Anuria	1 (0.4)	11 (9.6)	<0.001
Seizures	2 (0.7)	9 (7.8)	<0.001
Disturbance of consciousness	9 (3.3)	26 (22.6)	<0.001
Reduced responsiveness	2 (0.7)	10 (8.7)	<0.001
Agitation	9 (3.3)	17 (14.8)	<0.001
Limb dysfunction	0 (0.0)	4 (3.5)	0.008
Fasciculation/myoclonus	0 (0.0)	1 (0.9)	0.299
Blurred vision	0 (0.0)	3 (2.6)	0.026
Hoarseness	5 (1.9)	2 (1.7)	1.000
Dysarthria	0 (0.0)	3 (2.6)	0.026
Lung exudation	161 (59.6)	57 (49.6)	0.087
Pleural effusion	23 (8.5)	26 (22.6)	<0.001
Mediastinal emphysema	29 (10.7)	1 (0.9)	0.002
Pulmonary fibrosis	12 (4.4)	2 (1.7)	0.246
Admission laboratory findings, median (IQR)			
WBC, ×10^9/L	15.00 (10.79–19.93)	15.63 (10.12–22.10)	0.836
ALT, U/L	38.00 (16.30–209.10)	58.45 (16.75–197.98)	0.660
AST, U/L	36.70 (21.85–204.75)	42.85 (19.85–145.30)	0.689
CK, U/L	155.00 (77.00–422.50)	198.00 (76.00–1541.00)	0.016
CK-MB, U/L	20.50 (12.00–36.25)	28.20 (15.30–69.00)	<0.001
Creatinine, μmol/L	270.00 (121.50–470.45)	216.50 (98.00–333.75)	0.014
Amylase, U/L	102.00 (57.57–421.00)	88.75 (60.15–161.25)	0.057

PQ, paraquat; DQ, diquat; WBC, white blood cell; ALT, alanine aminotransferase; AST, aspartate aminotransferase; CK, creatine kinase; CK-MB, creatine kinase-MB. Continuous variables are presented as median (IQR) and were compared using the Mann- Whitney U test. Categorical variables are presented as *n* (%) and were compared using the chi-square test or Fisher’s exact test, as appropriate.

**Figure 2:**
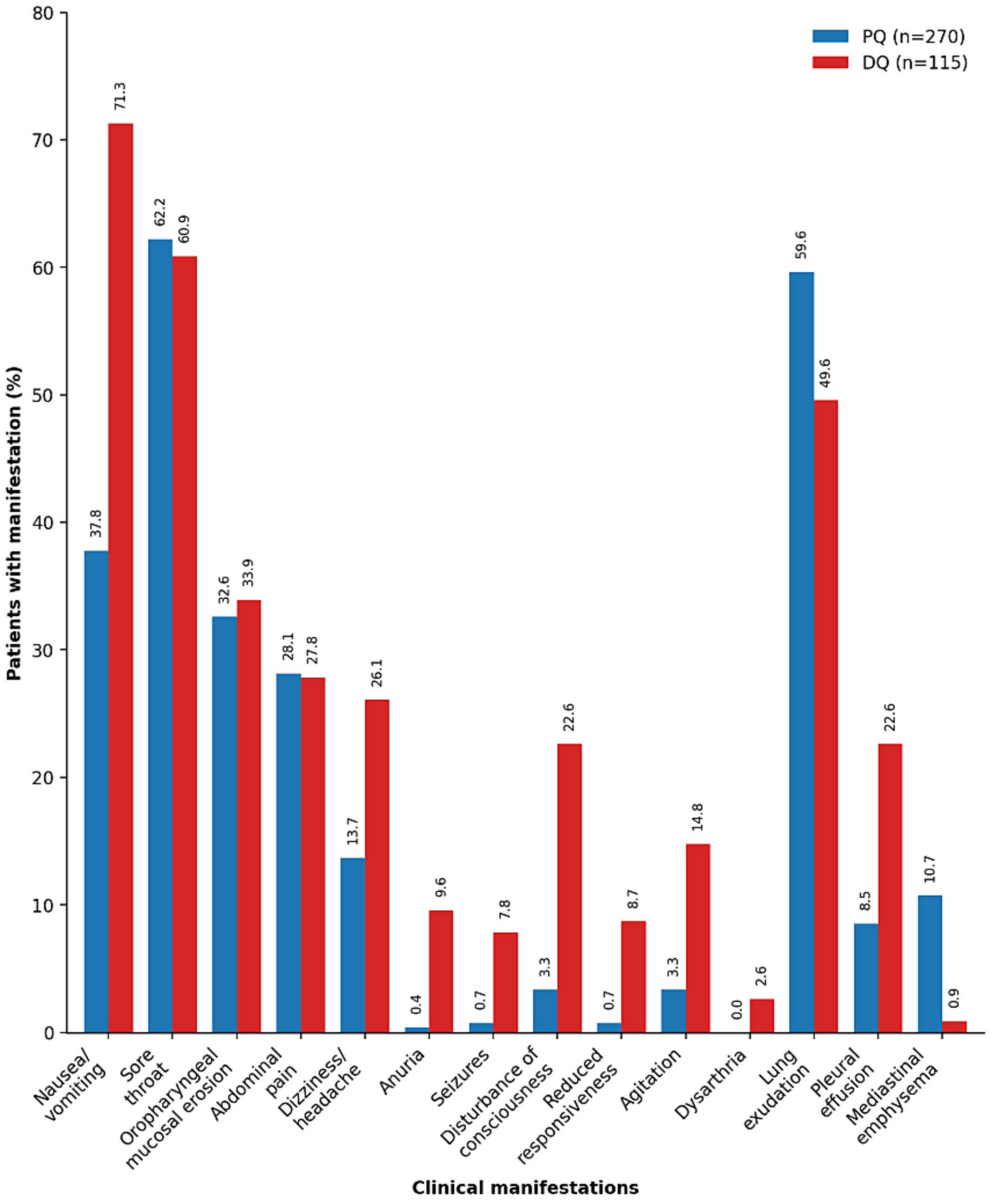
Percentage of patients with selected clinical manifestations in the PQ and DQ groups.

**Figure 3:**
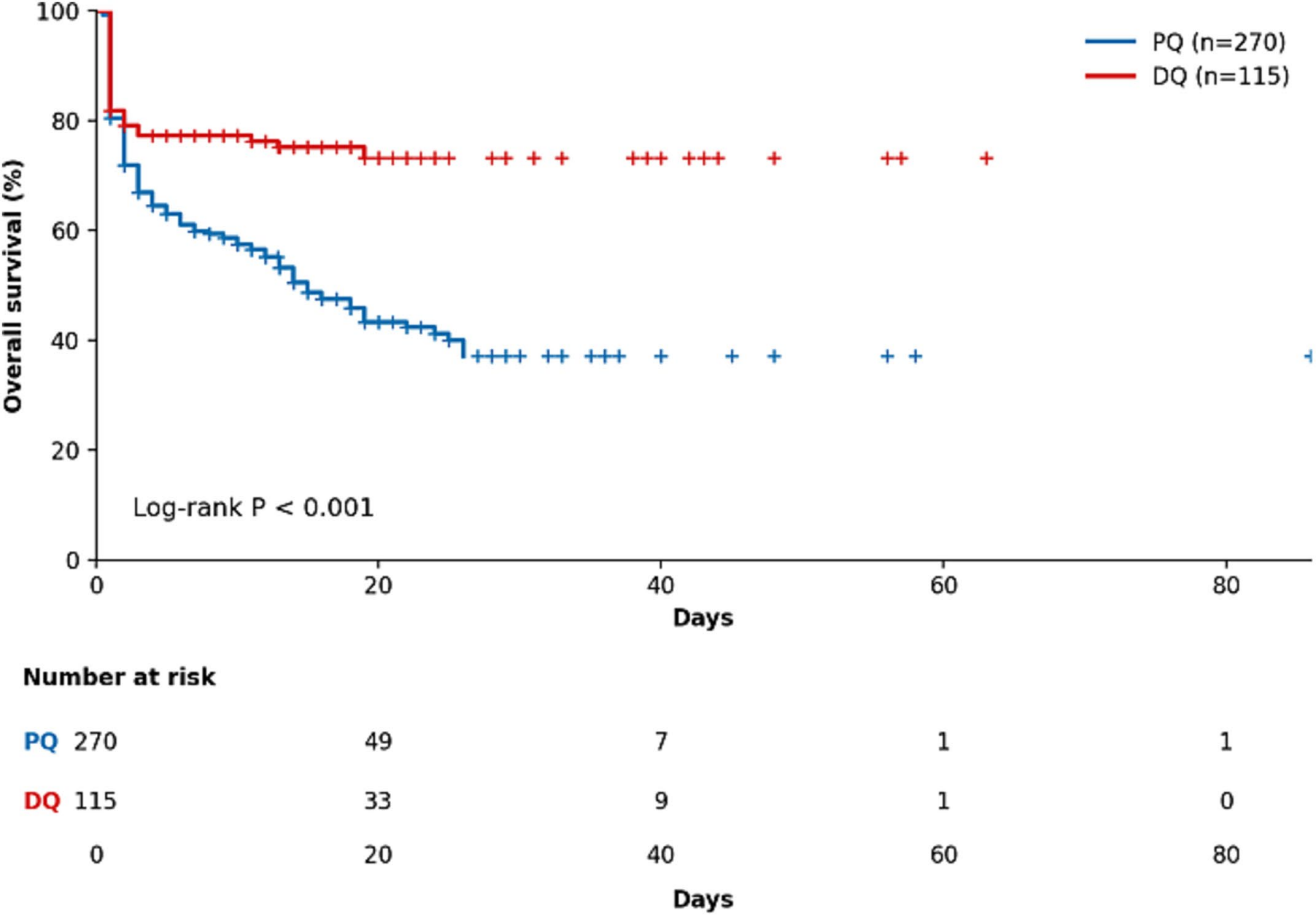
Kaplan–Meier survival curves for the PQ and DQ groups with numbers at risk.

Detailed frequencies of clinical manifestations are summarized in Table 1 and illustrated in Figure 2. Nausea/vomiting was common in both groups but occurred more frequently in DQ poisoning (71.3% vs. 37.8%, *p* < 0.001). DQ poisoning was also associated with a higher frequency of neurologic manifestations, including disturbance of consciousness (22.6% vs. 3.3%), seizures (7.8% vs. 0.7%), reduced responsiveness (8.7% vs. 0.7%), agitation (14.8% vs. 3.3%), and dysarthria (2.6% vs. 0%; all *p* ≤ 0.026). Anuria was markedly more frequent in the DQ group (9.6% vs. 0.4%, *p* < 0.001). By contrast, mediastinal emphysema was more common in PQ poisoning (10.7% vs. 0.9%, *p* = 0.002). Lung exudation was numerically more frequent in PQ poisoning but did not differ significantly between groups (59.6% vs. 49.6%, *p* = 0.087). Kaplan–Meier analysis demonstrated a significant difference in overall survival between the two groups (log-rank *p* < 0.001) (Figure 3).

### 3.1 Epidemiologic features and dominant fatal pathways

To further improve the clinical interpretability of the cohort, we reviewed the available records for additional epidemiologic and fatal-pathway information. Because this retrospective study spanned a long period and the documentation of exposure intent was not fully standardized across all cases, a formal quantitative comparison of accidental, intentional, and other exposure circumstances could not be performed reliably for the entire cohort. Nevertheless, the overall case pattern supports the continued clinical coexistence of both PQ and DQ poisoning, underscoring the need for toxin-specific evaluation rather than treating them as interchangeable bipyridyl intoxications.

Among non-survivors, the dominant fatal clinical pathways also appeared to differ between the two groups. Fatal PQ poisoning more commonly followed a pulmonary-dominant course, whereas DQ poisoning more often showed prominent neurologic deterioration, severe renal injury, or multiorgan dysfunction. Although adjudicated cause-of-death categories were not uniformly available for all cases, these observed differences were consistent with the broader between-group patterns seen in the presenting manifestations, laboratory findings, and survival analysis.

## 4 Discussion

This retrospective single-center study compared the clinical features, organ injury patterns, and in-hospital outcomes of acute paraquat (PQ) and diquat (DQ) poisoning and showed that these two bipyridyl herbicides should not be regarded as interchangeable toxic syndromes. Although PQ and DQ share structural similarity, the data in this cohort support distinct dominant patterns of toxicity (2, 3, 5, 7–12). PQ poisoning was associated with a higher crude in-hospital mortality and more pulmonary-dominant injury features, whereas DQ poisoning was characterized more often by neurologic manifestations, anuria, pleural effusion, and higher CK/CK-MB levels. Mechanistically, pulmonary fibrosis after PQ exposure has been associated with oxidative stress, mitochondrial injury, epithelial-cell senescence-derived exosomes, ferroptosis-related pathways, mitophagy, and hypoxia-driven fibroblast proliferation, which may help explain the pulmonary-dominant phenotype observed in severe PQ poisoning (29, 31, 33–35). These between-group differences are clinically relevant because they may influence early monitoring priorities, bedside risk assessment, and the timing of escalation of supportive care.

The higher crude in-hospital mortality observed in PQ poisoning is directionally consistent with the well-recognized lethality of PQ and with previous reports emphasizing the prognostic importance of early cardiopulmonary and metabolic abnormalities in severe poisoning (5–7, 13–15). In PQ poisoning, immune-cell alterations, toxicokinetic profiles, acute kidney injury, and pulmonary fibrotic progression have also been reported to be associated with disease severity and prognosis (27, 30, 32). However, this comparison should be interpreted cautiously. In addition to differences in age, reported ingestion amount, and time to presentation, this retrospective study was not designed to determine toxin-specific causal mortality differences, and no multivariable regression analysis was performed to adjust for potential confounding. Accordingly, our findings are best interpreted as demonstrating different crude clinical trajectories rather than proving that one toxin is intrinsically more lethal than the other under all conditions.

The admission laboratory findings further differentiated the two toxidromes. Higher creatinine levels in the PQ group suggested more prominent early renal impairment at presentation, whereas DQ poisoning more often showed anuria together with neurologic manifestations and higher CK/CK-MB levels, supporting more pronounced neuromuscular, neurologic, and systemic injury in severe DQ cases (8–12). These findings are clinically meaningful because early identification of the dominant organ injury pattern may help prioritize surveillance strategies. In practical terms, PQ poisoning may require particularly close attention to pulmonary progression and respiratory deterioration, whereas DQ poisoning may warrant intensified neurologic observation and renal monitoring from an earlier stage. Importantly, renal involvement may differ not only in severity but also in clinical pattern between the two groups; in our cohort, PQ was associated with higher admission creatinine, whereas DQ more often manifested with overt anuria. These observations are consistent with previous case-based and mechanistic studies showing that DQ poisoning can be accompanied by severe renal injury, glomerulotubular nephritis, neurologic injury, and multisystem dysfunction, and that PQ and DQ poisoning may involve partly distinct molecular pathways (23, 24, 26, 28).

The shorter hospital stay observed in PQ poisoning despite a higher crude mortality likely reflects rapid deterioration in a substantial proportion of severe PQ cases. By contrast, the longer hospital stay in DQ poisoning may reflect a longer window for organ support, continued neurologic observation, and management of evolving renal or multisystem injury. This difference further supports the view that PQ and DQ poisoning follow distinct clinical pathways after ingestion, even when both initially present as acute bipyridyl herbicide intoxication.

Beyond the descriptive differences in organ injury patterns, the therapeutic implications of early toxin recognition also deserve emphasis. Hemoperfusion remains one of the most commonly used extracorporeal elimination strategies in acute bipyridyl herbicide poisoning. Published evidence has suggested that hemoperfusion, particularly when combined with other supportive measures, may be associated with lower mortality in paraquat poisoning, although the underlying evidence is largely retrospective and heterogeneous (16). More recent reports have also discussed hemoperfusion combined with continuous renal replacement therapy and therapeutic plasma exchange as potentially useful adjunctive approaches in selected life-threatening cases (17, 18). For patients with catastrophic respiratory or cardiopulmonary failure, extracorporeal membrane oxygenation may serve as a rescue or bridge strategy in highly selected patients rather than a routine therapy, including use as a bridge to lung transplantation in end-stage toxic pulmonary injury (19, 20). Therefore, our findings should be interpreted as supporting earlier risk stratification and timely escalation of organ support, rather than as proving the efficacy of any specific extracorporeal modality.

Laboratory-based differential diagnosis between PQ and DQ poisoning also has practical clinical importance. Although both toxins belong to the bipyridyl herbicide class and may initially present with overlapping gastrointestinal and systemic symptoms, their subsequent organ injury trajectories differ substantially. In our cohort, PQ poisoning was more strongly linked to pulmonary-dominant injury and higher crude mortality, whereas DQ poisoning more often manifested with neurologic dysfunction, anuria, pleural effusion, and higher CK/CK-MB levels. Therefore, rapid toxin-specific identification may help clinicians tailor early monitoring priorities and supportive strategies. Recently reported diagnostic approaches combining colloidal gold immunochromatographic screening with hydrophilic interaction liquid chromatography have shown potential for rapid differentiation between paraquat and diquat poisoning and for blood toxin monitoring, particularly when the exposure history is unclear (21). From a clinical standpoint, this is important because the decision to intensify pulmonary surveillance, renal support, neurologic monitoring, or extracorporeal treatment may depend not only on poisoning severity but also on correct toxin identification at an early stage.

The epidemiologic pattern observed in this cohort also deserves attention. Although paraquat was banned in China in 2016, PQ poisoning continued to be encountered in clinical practice during the study period, while DQ poisoning emerged as an increasingly important clinical entity (1, 8, 9). This changing case pattern highlights the need to update diagnostic vigilance and management strategies for bipyridyl herbicide poisoning rather than continuing to view PQ as the sole dominant toxic exposure in this class. At the same time, the long study period raises the possibility of secular confounding, because referral pathways, product availability, formulation patterns, and supportive management may all have changed over time.

This study has several limitations. First, its retrospective single-center design introduces the possibility of selection bias and limits external generalizability. Second, patients transferred before outcome evaluation and those with incomplete records were excluded, which may have affected the final cohort. Third, the long study period spanned substantial changes in paraquat availability and clinical practice, introducing potential temporal confounding. Fourth, no multivariable logistic or Cox regression analyses were performed, and the crude mortality comparison may therefore be influenced by baseline differences between the two groups. Fifth, exposure-intent categories and formally adjudicated cause-of-death data were not uniformly documented across the full retrospective period, which limited more granular epidemiologic and cause-specific mortality comparisons. Finally, the absence of serial biomarker measurements limited our ability to evaluate the dynamic evolution of organ injury over time.

Overall, our data support a clinically important distinction between PQ and DQ poisoning. Rather than treating them as interchangeable bipyridyl intoxications, clinicians should recognize that PQ poisoning in this cohort more often followed a pulmonary-dominant and rapidly progressive course, whereas DQ poisoning more often involved neurologic dysfunction, renal complications, and prolonged supportive care needs. This distinction may help improve toxin-specific monitoring, guide escalation of organ support, and strengthen early bedside decision-making in severe herbicide poisoning.

## 5 Conclusion

In summary, paraquat and diquat poisoning should not be approached as interchangeable bipyridyl intoxications. In this retrospective single-center cohort, PQ poisoning showed a higher crude in-hospital mortality and a more pulmonary-dominant injury profile, whereas DQ poisoning more often presented with neurologic dysfunction, anuria, pleural effusion, and higher CK/CK-MB levels. Clinically, the management pathway should prioritize rapid toxin identification, prompt gastrointestinal decontamination and extracorporeal toxin removal when indicated, early pulmonary surveillance and respiratory support in PQ poisoning, and intensified neurologic and renal monitoring in DQ poisoning. Further multicenter studies with more standardized epidemiologic documentation and formal cause-of-death adjudication are needed to refine risk stratification and clarify how specific rescue strategies may improve outcomes.

## Data Availability

The original contributions presented in the study are included in the article/supplementary material, further inquiries can be directed to the corresponding author.
